# Low Level of PALMD Contributes to the Metastasis of Uveal Melanoma

**DOI:** 10.3389/fonc.2022.802941

**Published:** 2022-04-13

**Authors:** Min-Yun Cai, Yue-Li Xu, Hua Rong, Hai Yang

**Affiliations:** ^1^Department of Ophthalmology, Shanghai East Hospital, Shanghai, China; ^2^Department of Ophthalmology, Shanghai Jiangong Hospital, Shanghai, China

**Keywords:** Uveal melanoma, metastasis, Palmd, ZNF263, MMP

## Abstract

Uveal melanoma (UM) is a highly aggressive disease. There is an urgent need to develop the metastasis prediction markers of UM. This study aims to detect the key role of PALMD in UM metastasis. Transcriptome sequencing results of 2 sets of UM metastatic samples (GSE22138 and GSE156877) were downloaded from the Gene Expression Omnibus (GEO), and 18 overlapping differentially expressed genes were screened out, including PALMD. PALMD was significantly underexpressed in metastatic UM tissue. Low expression of PALMD was associated with poor prognosis in UM patients. The decreased expression of PALMD promoted the invasion and migration of 92-1 and Mel270 cells, while the high expression of PALMD inhibited the invasion and migration of UM cells. Furthermore, the levels of matrix metallopeptidase (MMP) 2 and MMP9 increased after transfection of siRNAs specifically targeting PALMD, whereas the levels of MMP2 and MMP9 were decreased after PALMD overexpression. However, PALMD did not affect the proliferation of UM cells. In addition, ZNF263 promoted the transcription of PALMD through the putative binding sequence using the JASPAR database, luciferase reporter gene analysis and chromatin immunoprecipitation assay. In summary, the expression of PALMD regulated by ZNF263 plays an important role in UM metastasis.

## Introduction

Uveal melanoma (UM) is the most common primary eye cancer in adults, which originates from the melanocytes of the eye (1). Through radiotherapy, UM has good local tumor control. However, UM is a highly aggressive disease with a moderate risk of metastasis. After the diagnosis of metastasis, the patient’s survival time is less than 12 months (2). Up to 50% of patients have metastatic deaths (3). Therefore, there is an urgent need to develop the metastasis prediction markers of UM.

Through genome-wide and transcriptome analysis, *PALMD* is considered to be the causative gene of calcific aortic stenosis (CAS) (4–6). *PALMD* alleles in valve tissue confer disease susceptibility by reducing the level of PALMD mRNA (7). The *PALMD* gene is located at 1p21.2 and encodes Palmdelphin (PALMD) protein (8). The study has suggested that PALMD protein is a regulator of actin polymerization (9). Lower level of PALMD promotes the polymerization of actin, the activation of myocardial-related transcription factors, and the development of fibrosis programs, which are the key pathobiological processes in the CAS progress (9). The PALMD protein belongs to the paralemmin family, which is involved in the control of cell morphology and plasma membrane motility (10). PALMD has also been found to have a specific role in the morphological changes in the differentiation process of myoblasts (11). The study has also shown that membrane-bound PALMD promotes the proliferation and morphogenesis of basal cells in mammalian neocortex through integrin signals (12). However, no studies have reported the role of PALMD in diseases other than CAS.

This study will detect the key role of PALMD in UM metastasis by using transcriptome analysis and cell experiments.

## Material and Methods

### Raw Biological Microarray Data and Elucidation of Differentially Expressed Genes

2 transcriptome sequencing results were downloaded from the Gene Expression Omnibus (GEO) database (13), GSE22138 (34 UM tissue samples with liver metastases and 29 UM tissues without metastasis, Affymetrix GPL570 platform) and GSE156877 (4 UM tissue samples with early metastasis and 4 without metastasis, Affymetrix GPL23126 platform). Corresponding gene probes were converted into symbols according to the annotation information in the platform. DEGs between metastatic and non-metastatic UM samples were screened and determined using GEO2R. DEGs were filtered using parameters such as adjusted P-values (adj.P), Benjamini, and fold changes. Probe sets without corresponding gene symbols were removed; genes with multiple probe sets were averaged. Log2 (Fold change)>1 or <-1 & P<0.01 were considered statistically significant.

### Survival Analysis

The correlation between overall survival (OS) or disease-free survival (DFS) and PALMD mRNA levels in 78 UM patients was analyzed by Gene Expression Profiling Interactive Analysis (GEPIA) web service (14). UM patients were grouped by median PALMD mRNA levels. The Kaplan-Meier method, 95% confidence interval (95% CI) and log-rank test were used to analyze PALMD-related survival.

### Cell Culture and Transfection

UM cell lines (92.1 and Mel270) were purchased from the cell banks of Chinese Academy of Sciences (Shanghai, China). All cells were cultured in high-glucose DMEM (Invitrogen) with 0.1 mg/ml streptomycin, 100 U/ml penicillin, and 10% (v/v) FBS in a humidified cell incubator at 37°C and 5% CO_2_. siRNAs targeting PALMD and ZNF263, PALMD and ZNF263 pcDNA3.1 overexpression plasmids were synthesized from GeneChem company (Shanghai), and transfected into cells using Lipofectamine 2000 (Invitrogen, USA) according to the manufacturer’s protocol.

### Western Blot

Cells were lysed with RIPA lysate, and total protein was collected with 12,000 g centrifugation at 4°C for 10 min. The protein concentration was measured by BCA kit (Pierce). The protein was separated by SDS-polyacrylamide gel electrophoresis and transferred to a PVDF membrane. After blocking with 5% skimmed milk for 90 min, the membrane was incubated with the primary antibody overnight at 4°C, and then performed the secondary antibody at room temperature for 2 h. The bands were visualized using an enhanced chemiluminescence system (Millipore, USA). The primary antibodies used in this study are as follows: PALMD (Proteintech; 1:2000; 16531-1-AP), ZNF263 (Abcam; 1:1000; ab129100) and GAPDH (Proteintech; 1:5000; 60004-1-Ig).

### Transwell Assay

The insert (Millipore) was put into a 24-well plate and Matrigel (BD Biosciences) coated the membrane of insert. 600 μl of medium containing 2% FBS was added to the lower chamber. 10,000 cells were suspended in 200 μl serum-free medium and added to the upper chamber. After culturing for 24 h, the medium and cells in the upper chamber were removed, and washed with PBS. The cells invaded under the membrane were fixed with methanol and stained with crystal violet for 15 min. Subsequently, the cells were observed and counted under a microscope.

### Scratch Assay

The cells were cultured to form a confluent cell monolayer, and a sterile 10 μl pipette tip mechanically created a linear wound. After washing the exfoliated cells with PBS, cells were cultured for 24 h. The average distance of wound healing was measured with a microscope calibrated with an eyepiece micrometer.

### ELISA

Human matrix metallopeptidase (MMP) 2 (ab267813) and MMP9 (ab246539) ELISA Kits were purchased from Abcam, and performed according to the manufacturer’s protocol.

### CCK-8 Assay

Cells were seeded into each well of a 96-well plate and cultured for 72 h. Subsequently, the medium of each well was removed, and washed once with PBS. 10% CCK-8 reagent was added to each well and incubated for 2 h. A microplate reader (BioTek, USA) was used to detect the absorbance of each well at 450 nm.

### Transcription Factor Prediction

Enter the NCBI database and find the potential promoter sequence of the target gene *PALMD* (Chr1: 99644113-99646113). Enter the UCSC database (combined with JASPAR), enter the potential promoter region of the *PALMD* gene, and obtain the potential transcription factors that bind to the promoter region. Based on transcription factors and scores, ZNF263 was selected. Using JASPAR, predict the binding site sequence of ZNF263 in the promoter region of the target gene.

### Luciferase Reporter Assay

A firefly luciferase reporter plasmid containing wild-type (WT) or mutant (MUT) promoter sequences and pcDNA3.1-ZNF263 plasmid were co-transfected into 92-1 cells. After 48 h of incubation, the luciferase activity was determined according to the manufacturer’s protocol (Promega, E1910).

### Chromatin Immunoprecipitation Assay

92-1 cells over-expressed ZNF263 were fixed and crosslinked in 1% formaldehyde for 10 min at 37°C and incubated with protease inhibitors. Chromatin was isolated and enzymatically fragmented using an EZ-Zyme Chromatin Prep Kit (17375, Merck). Rabbit anti-ZNF263 antibody (21326-1-AP, Proteintech) or nonspecific IgG (1:200, Sigma) was used to precipitate DNA crosslinked with the ZNF263. The immunoprecipitated promoter fragment containing the ZNF263 response element was probed by PCR using primers targeting the regulatory region of PALMD gene and visualized by agarose gel electrophoresis.

### Statistical Analysis

GraphPad Prism 8 was used for statistical analyses, and all data were expressed as mean ± standard deviation (SD). Student t-test was used to compare data between two groups, and P<0.05 was considered to be statistically significant. The experiments *in vitro* were repeated at least 3 times.

## Results

### DEGs in UM Metastasis

**Figure 1:**
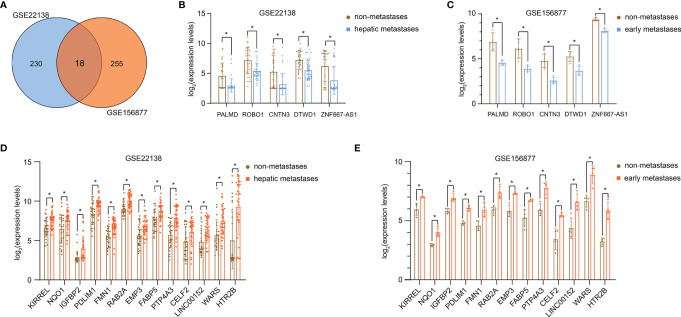
DEGs in UM metastasis. **(A)** Venn diagram of DEGs from GSE22138 and GSE156877. The expression of 5 genes significantly down-regulated in GSE22138 **(B)** and GSE156877 **(C)**. The expression of 13 genes significantly up-regulated in GSE22138 **(D)** and GSE156877 **(E)**. *P < 0.05.

248 DEGs were screened on the GSE22138, 273 DEGs were screened on the GSE156877, and 18 overlapping DEGs were screened out (**Figure 1A**). Among them, the expression of 5 genes was significantly down-regulated (**Figures 1B, C**), and the expression of 13 genes was significantly up-regulated (**Figures 1D, E**) in the metastatic UM tissue.

**Figure 2:**
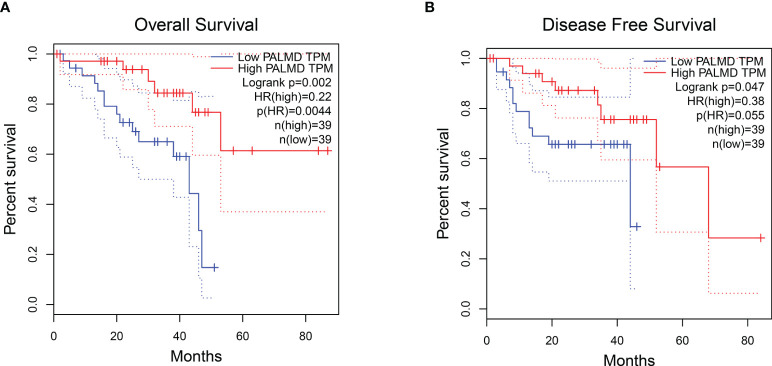
UM patients with high expression of PALMD have a better prognosis. The **(A)** overall survival and **(B)** disease-free survival of UM patients with low or high PALMD expression. Data from the GEPIA.

According to the literature review, other DEGs have been extensively studied in tumors or other types of diseases (**Supplementary Table 1**), excluding PALMD. PALMD is considered to be a target of aortic stenosis, and there are few studies. Furthermore, data from the GEPIA online analysis website (14) showed that the expression level of PALMD was closely related to the OS (**Figure 2A**) and DFS (**Figure 2B**) of UM patients. Patients with high expression of PALMD had a better prognosis (**Figure 2**). Therefore, this study mainly explores the role of PALMD in UM metastasis.

### Low Level of PALMD Is Beneficial to the Motility and Metastasis of UM Cells

**Figure 3:**
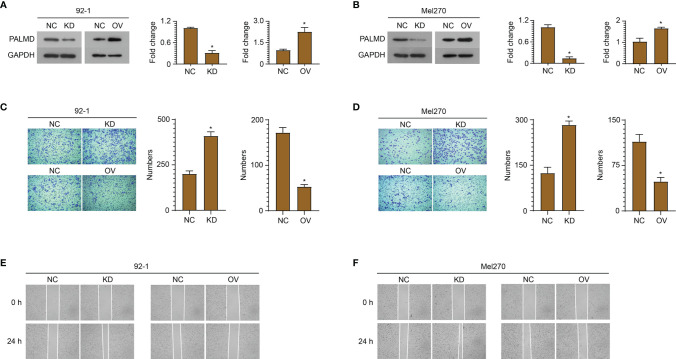
Low level of PALMD is beneficial to the motility and metastasis of UM cells. siRNA specifically targeting PALMD or pcDNA3.1 overexpression plasmid was transfected into 92-1 **(A)** and Mel270 **(B)** cells to down- (KD) or up- (OV) regulate the expression of PALMD. After transfection, the invasion of 92-1 **(C)** and Mel270 **(D)** cells was measured using Transwell assay, the migration of 92-1 **(E)** and Mel270 **(F)** cells was detected using scratch assay. *P < 0.05.

**Figure 4:**
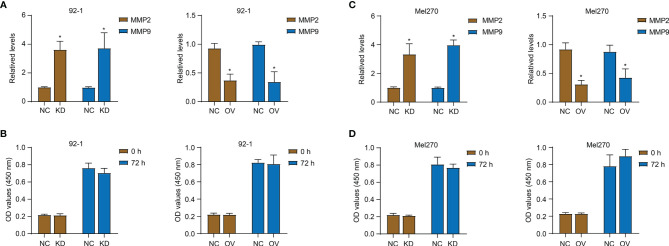
PALMD on MMP expression and proliferation of UM cells. After transfection of siRNA specifically targeting PALMD (KD) or pcDNA3.1 overexpression plasmid (OV), the MMP2 and MMP9 expression of 92-1 **(A)** and Mel270 **(B)** cells was measured with ELISA, the proliferation of 92-1 **(C)** and Mel270 **(D)** cells was detected with CCK-8 assay. *P < 0.05.

In order to explore the role of PALMD in the motility and metastasis of UM cells, siRNA (si-PALMD) specifically targeting PALMD (KD) or pcDNA3.1 PALMD overexpression plasmid (OV) was transfected into 92-1 and Mel270 cells to down- or up- regulate the expression of PALMD (**Figures 3A, B**). The result of Transwell experiment showed in **Figures 3C, D**, compared with the NC group, the number of cells passing through the Matrigel in the KD group was significantly increased, while the number of cells passing through the Matrigel in the OV group was significantly decreased. Furthermore, the result of scratch experiment showed in **Figures 3E, F**, compared with the NC group, the rate of wound healing in the KD group was faster, while that in the OV group was slower. In addition, we also used ELISA to detect the expression levels of MMP2 and MMP9. The results showed that after transfection of si-PALMD, the levels of MMP2 and MMP9 increased, while the transfection of PALMD overexpression plasmid reduced the MMP2 and MMP9 levels (**Figures 4A, B**). These results all indicate that low levels of PALMD are beneficial to the motility and metastasis of UM cells.

### PALMD Does Not Affect the Proliferation of UM Cells

In addition, we used the CCK-8 experiment to detect the effect of PALMD on cell proliferation (**Figures 4C, D**). As shown in **Figures 4C, D**, 72 h after down- or up- regulating PALMD, there was no significant difference in the absorbance values at 450 nm between the NC and experimental groups.

### ZNF263 Is Responsible for the Transcriptional Regulation of PALMD

**Figure 5:**
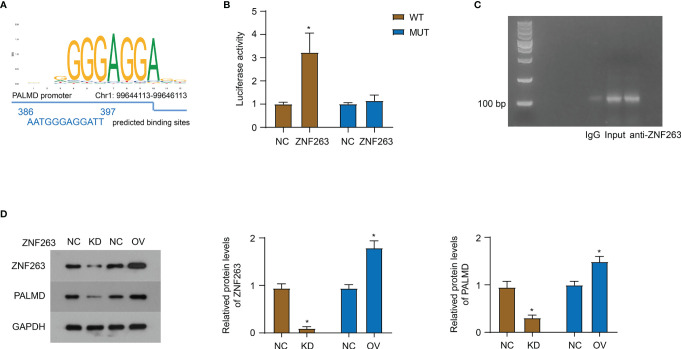
ZNF263 is responsible for the transcriptional regulation of PALMD. **(A)** ZNF263 was predicted to be a transcription factor binding to the PALMD promoter, and the binding site was AATGGGAGGATT. Data from JASPAR databases. **(B)** Luciferase reporter assay in 92-1 cells. **(C)** PCR and agarose gel amplification and detection of DNA sequences bound to the ZNF263 protein in 92-1 cells overexpressed ZNF263. **(D)** The effect of ZNF263 down- or up- regulated expression on PALMD protein expression was detected in 92-1 cells with western blot. *P < 0.05.

Using the UCSC and JASPAR databases, ZNF263 was predicted to be a transcription factor binding to the PALMD promoter, and the binding site was AATGGGAGGATT (**Figure 5A**). Subsequently, we conducted a luciferase experiment and co-transfected the pGL4.20 plasmid containing wild-type (WT) or mutant (MUT) binding site and ZNF263 overexpression plasmid into 92-1 cells. The results (**Figure 5B**) showed that ZNF263 activated the transcription of luciferase through the putative binding sequence. Furthermore, ChIP experiment verified the direct binding of ZNF263 to the *PALMD* promoter in 92-1 cells (**Figure 5C**). In addition, knockdown of ZNF263 down-regulated the PALMD expression, while exogenous expression of ZNF263 up-regulated the expression of PALMD in 92-1 cells (**Figure 5D**).

## Discussion

UM is an aggressive malignancy, and up to half of UM eventually metastasizes to other organs. Currently, there is no effective treatment for metastatic UM. Once metastasis occurs, disease-related death typically occurs within a year. The metastatic spread of cancer cells is a complex multistep process, and understanding the molecular mechanisms that initiate and promote UM metastasis is critical for the development of successful treatments. Therefore, in recent years, different researchers have made efforts to explore the mechanisms related to UM metastasis from the aspects of genomic mutation driver (15), epigenetic mechanism driver (16), altered transcriptomics (17) and altered proteomics (18). Currently, only limited results have been achieved. The Gαq pathway mutation and the increase in chromosome arm 8q are elucidated as common genomic evolutionary pathways applicable to most metastatic UM (15). Mutually exclusive gain-of-function mutations in members of the Gαq signaling pathway (GNAQ, GNA11, CYSLTR2, and PLCB4) represent initiating events present in more than 95% of UM (19). Aberrant methylation of *BAP1* and *SF3B1* is thought to be associated with UM metastasis (16). Furthermore, transcriptional analysis of metastatic UM nominates NRP1 as a predicted target (17). Jang et al. identify 32 proteins capable of predicting metastatic and non-metastatic UM with 93% discriminative accuracy (18).

In the present study, we extracted 18 transcripts with significantly different levels in metastatic UM using public transcriptome sequencing information. However, whether these 18 transcripts can be predictive targets for UM metastasis requires data analysis with an expanded clinical sample size. The arrays analyzed in this study contained small sample sizes. In addition, the pathological information and prognosis of patients were also lacking, and the correlation analysis between pathology or prognosis and transcripts cannot be performed. The genomic mutations, epigenetic regulation, post-transcriptional regulation, protein expression patterns, subcellular localization, and biological roles of these 18 genes in cancer cell progression deserve careful consideration. Furthermore, similar to our methods, Xu et al. also download three microarray datasets (GSE22138, GSE27831 and GSE73652) for patients with primary or metastatic UM from GEO, and identify 103 DEGs as candidate prognostic biomarkers of metastatic UM, and consider SCD5, SPTBN1, FABP5, SQLE, PTPLA (HACD1), and CDC25B as independent prognostic factors for UM (20). The microarray datasets selected for the two articles are different, and we used more stringent screening criteria: log2 (Fold change)>1 or <-1 & P<0.01. Xu et al. consider the absolute value of Log2FC (fold change) >1.00 and P value <0.05 to be statistically significant. In addition, unlike Xu’s wish to obtain several candidate prognostic biomarkers for metastatic UM through comprehensive analysis, we prefer to focus on the specific role of a specific gene/protein in the progression of UM through comprehensive analysis.

In this study, we mainly focused on PALMD. We found that PALMD was significantly underexpressed in metastatic UM tissues. Low levels of PALMD were closely associated with poor prognosis in UM patients. *In vitro* cell experiments also showed that low level of PALMD favored the invasion and migration of UM cells, and high expression of PALMD inhibited the migration of UM cells. These results indicate the importance of low expression of PALMD in the process of UM metastasis. However, the results of the CCK-8 assay indicated that PALMD expression levels had no effect on the proliferation of UM cells. Although one study suggests that PALMD is a pro-apoptotic gene induced by p53 in a specific way of phosphorylated serine-46 (21). In addition, one study has reported that emodin inhibits the growth of liver cancer cells, and 15 representative genes such as PALMD (down-regulated) are considered to be related to the response to emodin treatment (22). The tissue-specific function of PALMD and the specific mechanism of its effect still need to be further explored.

Furthermore, we used bioinformatics analysis, dual luciferase experiments and ChIP to find that ZNF263 can acted as a transcription factor of PALMD to promote its transcription. According to reports, ZNF263 is a key transcription factor for cholangiocarcinoma (23), gastric cancer (24), and hepatocellular carcinoma (25). However, these studies perform comprehensive analyses of cancer-related gene expression datasets in the GEO database, yielding key transcription factors differentially expressed in cancer, including ZNF263. Indeed, to our knowledge, no studies have reported the role of ZNF263 in cancer initiation and progression, its correlation with cancer-related events, and the exact regulatory genes. This study is the first to report the exact regulatory gene of ZNF263, PALMD, and its binding site. In addition, Wu et al. believe that the tumor suppressor gene (TSG) may have evolved specific characteristics that promote its tumor suppressor function, including high CpG dinucleotide frequency and ZNF263 binding element (26). This seems to illustrate the pivotal role of PALMD as TSG in the occurrence and development of UM. However, the mechanism of dysregulated expression of PALMD in UM still needs to be further explored.

The search for predictive markers of UM metastasis has important implications. This analysis identified 18 genes with predictive potential for UM metastasis. In addition, we found that the expression of PALMD regulated by ZNF263 plays an important role in UM metastasis. PALMD is a tumor suppressor gene that plays a key role in the progression of UM metastasis. In the future, we will mainly focus on how the expression of PALMD is dysregulated in UM progression and whether it is related to ZNF263.

## Data Availability Statement

The original contributions presented in the study are included in the article/**Supplementary Material**. Further inquiries can be directed to the corresponding author.

## Author Contributions

HY have made substantial contributions to the conception and design of the work. Each author has made substantial contributions to the acquisition, analysis, and interpretation of data. All authors contributed to the article and approved the submitted version.

## Conflict of Interest

The authors declare that the research was conducted in the absence of any commercial or financial relationships that could be construed as a potential conflict of interest.

## Publisher’s Note

All claims expressed in this article are solely those of the authors and do not necessarily represent those of their affiliated organizations, or those of the publisher, the editors and the reviewers. Any product that may be evaluated in this article, or claim that may be made by its manufacturer, is not guaranteed or endorsed by the publisher.
