# Supplementary material for: Low Level of PALMD Contributes to the Metastasis of Uveal Melanoma
Source: Front Oncol. 2022 Apr 13;12:802941. doi: 10.3389/fonc.2022.802941 (PMC9043551; doi:10.3389/fonc.2022.802941)
Supplement: Supplementary file 1 [file Table_1.docx]

|  | UM | other melanomas | other tumors | other diseases |
| --- | --- | --- | --- | --- |
| PALMD | n/a | n/a | n/a | calcific aortic valve stenosis^1-3^ |
| ROBO1 | metastasis^4^ | reduce invasion^5^  angiogenesis^6^ | Multiple myeloma^7^  Bladder cancer^8^  NSCLC^9^  …… | Hirschsprung disease^10^  Callosal Dysgenesis^11^  cardiac fibrosis^12^  …… |
| CNTN3 | n/a | n/a | renal cell carcinomas^13^  glioblastoma multiforme^14^  colorectal cancer^15^  …… | neurodevelopmental delay^16,17^  Male infertility^18^  Acute aortic dissection^19^  …… |
| DTWD1 | n/a | hypoxia^20^ | cell renal cell carcinoma^21^  gastric cancer^22^ | bipolar disorder^23^ |
| ZNF667-AS1 | n/a | inhibit proliferation and invasion^24^ | bladder cancer^25^  ovarian cancer^26^  oral squamous cell carcinoma^27^  …… | rheumatoid arthritis^28^  spinal cord injury^29^ |
| KIRREL | n/a | prognosis^30^ | breast cancer^31^  gastric cancer^32^  pancreatic cancer^33^  …… | mycoplasma pneumoniae^34^ |
| NQO1 | n/a | redox cycle  metabolize  metastasis  apoptosis^35^ | colorectal cancer^36^  gastric cancer^37^  breast cancer^38^  …… | acute kidney injury^39^  spinal cord injury^40^  Alzheimer's disease^41^  …… |
| IGFBP2 | n/a | regulate PD-L1 expression^42^  apoptosis^43^  BRAF V600E resistance^44^ | glioma^45^  colorectal cancer^46^  osteosarcoma^47^  …… | cardiometabolic dysfunction^48^  obesity^49^  diabetes^50^  …… |
| PDLIM1 | n/a | n/a | ovarian cancer^51^  glioma^52^  chronic myelogenous leukaemia^53^  …… | diabetic retinopathy^54^  Cardiovascular Disease^55^  Friedreich's Ataxia^56^  …… |
| FMN1 | n/a | n/a | pancreatic cancer^57^  colorectal cancer^58^ | Cenani-Lenz syndrome^59^  Pseudoexfoliation syndrome^60^  schizophrenia^61^  …… |
| RAB2A | n/a | n/a | glioblastoma^62^  bladder cancer^63^  breast cancer^64^  …… | osteoarthritis^65^  autism and schizophrenia^66^  Alzheimer's disease^67^  …… |
| EMP3 | aggressiveness^68^ | n/a | lung cancer^69^  glioma^70^  acute myeloid leukemia^71^  …… | keratoconus^72^ |
| FABP5 | prognosis^73,74^ | high expression^75^  apoptosis^76^  cisplatin sensitivity^77^ | lung adenocarcinoma^78^  gastric cancer^79^  breast cancer^80^  …… | type 2 Diabetes Mellitus^81^  psoriasis^82^  cerebral ischemia-reperfusion^83^  …… |
| PTP4A3 | liver metastasis^84^  aggressiveness^85,86^ | stem cell differentiation^87^  chemotherapy^88^  migration^89^  …… | multiple myeloma^90^  ovarian cancer^91^  colorectal cancer^92^  …… | Alzheimer disease^93^ |
| CELF2 | prognosis^94^ | n/a | prostate cancer^95^  nasopharyngeal carcinoma^96^  breast cancer^97^  …… | Alzheimer's disease^98^ |
| LINC00152 | n/a | n/a | glioma^99^  acute myeloid leukemia^100^  breast cancer^101^  …… | rheumatoid arthritis^102^ |
| WARS | prognosis^94^  progression^103^ | n/a | n/a | n/a |
| HTR2B | high expression^104,105^  metastasis^4^ | n/a | colorectal cancer^106^  pancreatic cancer^107^  ovarian cancer^108^  …… | atrial fibrillation^109^  age-related osteoarthritis^110^  constipation^111^  …… |

Supplementary Table 1: Research status of differentially expressed genes in UM metastasis tissues. For more than 3 diseases, only the first three are displayed.

**References**

1. Chignon A, Rosa M, Boulanger MC, et al. Enhancer-associated aortic valve stenosis risk locus 1p21.2 alters NFATC2 binding site and promotes fibrogenesis. *iScience.* 2021;24(3):102241.

2. Li Z, Gaudreault N, Arsenault BJ, Mathieu P, Bosse Y, Theriault S. Phenome-wide analyses establish a specific association between aortic valve PALMD expression and calcific aortic valve stenosis. *Commun Biol.* 2020;3(1):477.

3. Theriault S, Dina C, Messika-Zeitoun D, et al. Genetic Association Analyses Highlight IL6, ALPL, and NAV1 As 3 New Susceptibility Genes Underlying Calcific Aortic Valve Stenosis. *Circ Genom Precis Med.* 2019;12(10):e002617.

4. Demirci H, Reed D, Elner VM. Tissue-based microarray expression of genes predictive of metastasis in uveal melanoma and differentially expressed in metastatic uveal melanoma. *J Ophthalmic Vis Res.* 2013;8(4):303-307.

5. Grossmann AH, Yoo JH, Clancy J, et al. The small GTPase ARF6 stimulates beta-catenin transcriptional activity during WNT5A-mediated melanoma invasion and metastasis. *Sci Signal.* 2013;6(265):ra14.

6. Wang B, Xiao Y, Ding BB, et al. Induction of tumor angiogenesis by Slit-Robo signaling and inhibition of cancer growth by blocking Robo activity. *Cancer Cell.* 2003;4(1):19-29.

7. Bianchi G, Czarnecki PG, Ho M, et al. ROBO1 Promotes Homing, Dissemination, and Survival of Multiple Myeloma within the Bone Marrow Microenvironment. *Blood Cancer Discov.* 2021;2(4):338-353.

8. Feng L, Fu D, Gao L, Cheng H, Zhu C, Zhang G. Circular RNA_0001495 increases Robo1 expression by sponging microRNA-527 to promote the proliferation, migration and invasion of bladder cancer cells. *Carcinogenesis.* 2021.

9. Zheng J, Li X, Cai C, Hong C, Zhang B. MicroRNA-32 and MicroRNA-548a Promote the Drug Sensitivity of Non-Small Cell Lung Cancer Cells to Cisplatin by Targeting ROBO1 and Inhibiting the Activation of Wnt/beta-Catenin Axis. *Cancer Manag Res.* 2021;13:3005-3016.

10. Kong M, Zhou T, Xiang B. Expression of the axon guidance factor Slit2 and its receptor Robo1 in patients with Hirschsprung disease: An observational study. *Medicine (Baltimore).* 2021;100(33):e26981.

11. Woodring TS, Mirza MH, Benavides V, et al. Uncertain, Not Unimportant: Callosal Dysgenesis and Variants of Uncertain Significance in ROBO1. *Pediatrics.* 2021;148(1).

12. Liu Y, Yin Z, Xu X, et al. Crosstalk between the activated Slit2-Robo1 pathway and TGF-beta1 signalling promotes cardiac fibrosis. *ESC Heart Fail.* 2021;8(1):447-460.

13. Yusenko MV, Nagy A, Kovacs G. Molecular analysis of germline t(3;6) and t(3;12) associated with conventional renal cell carcinomas indicates their rate-limiting role and supports the three-hit model of carcinogenesis. *Cancer Genet Cytogenet.* 2010;201(1):15-23.

14. Zhu YF, Guo YB, Zhang HY, et al. Prognostic significance of contactin 3 expression and associated genes in glioblastoma multiforme. *Oncol Lett.* 2019;18(2):1863-1871.

15. Zhou J, Xie Z, Cui P, et al. SLC1A1, SLC16A9, and CNTN3 Are Potential Biomarkers for the Occurrence of Colorectal Cancer. *Biomed Res Int.* 2020;2020:1204605.

16. Morales C, Mademont-Soler I, Armengol L, et al. Characterization of a 5.8-Mb interstitial deletion of chromosome 3p in a girl with 46,XX,inv(7)dn karyotype and phenotypic abnormalities. *Cytogenet Genome Res.* 2009;125(4):334-340.

17. Bouyain S, Watkins DJ. The protein tyrosine phosphatases PTPRZ and PTPRG bind to distinct members of the contactin family of neural recognition molecules. *Proc Natl Acad Sci U S A.* 2010;107(6):2443-2448.

18. Congras A, Yerle-Bouissou M, Pinton A, et al. Sperm DNA methylation analysis in swine reveals conserved and species-specific methylation patterns and highlights an altered methylation at the GNAS locus in infertile boars. *Biol Reprod.* 2014;91(6):137.

19. Zheng J, Guo J, Huang L, et al. Genetic diagnosis of acute aortic dissection in South China Han population using next-generation sequencing. *Int J Legal Med.* 2018;132(5):1273-1280.

20. Loftus SK, Baxter LL, Cronin JC, Fufa TD, Program NCS, Pavan WJ. Hypoxia-induced HIF1alpha targets in melanocytes reveal a molecular profile associated with poor melanoma prognosis. *Pigment Cell Melanoma Res.* 2017;30(3):339-352.

21. Yang F, Zhao J, Luo X, et al. Transcriptome Profiling Reveals B-Lineage Cells Contribute to the Poor Prognosis and Metastasis of Clear Cell Renal Cell Carcinoma. *Front Oncol.* 2021;11:731896.

22. Ma Y, Yue Y, Pan M, et al. Histone deacetylase 3 inhibits new tumor suppressor gene DTWD1 in gastric cancer. *Am J Cancer Res.* 2015;5(2):663-673.

23. Budde M, Friedrichs S, Alliey-Rodriguez N, et al. Efficient region-based test strategy uncovers genetic risk factors for functional outcome in bipolar disorder. *Eur Neuropsychopharmacol.* 2019;29(1):156-170.

24. Yan K, Wang Y, Shao Y, Xiao T. Gene Instability-Related lncRNA Prognostic Model of Melanoma Patients via Machine Learning Strategy. *J Oncol.* 2021;2021:5582920.

25. Zheng Z, Lai C, Li W, Zhang C, Ma K, Yao Y. Identification of a Novel Glycolysis-Related LncRNA Signature for Predicting Overall Survival in Patients With Bladder Cancer. *Front Genet.* 2021;12:720421.

26. Burdennyy AM, Filippova EA, Ivanova NA, et al. Hypermethylation of Genes in New Long Noncoding RNA in Ovarian Tumors and Metastases: A Dual Effect. *Bull Exp Biol Med.* 2021;171(3):370-374.

27. Zhao J, Cui Z, Dong Z, Niu Y, Yang K, Han H. IncRNA ZNF667-AS1 Suppresses Epithelial Mesenchymal Transformation by Targeting TGF-beta1 in Oral Squamous Cell Carcinoma. *Clin Lab.* 2021;67(7).

28. Zhuo Q, Wei L, Yin X, et al. LncRNA ZNF667-AS1 alleviates rheumatoid arthritis by sponging miR-523-3p and inactivating the JAK/STAT signalling pathway. *Autoimmunity.* 2021:1-9.

29. Li JW, Kuang Y, Chen L, Wang JF. LncRNA ZNF667-AS1 inhibits inflammatory response and promotes recovery of spinal cord injury via suppressing JAK-STAT pathway. *Eur Rev Med Pharmacol Sci.* 2018;22(22):7614-7620.

30. Lundgren S, Fagerstrom-Vahman H, Zhang C, et al. Discovery of KIRREL as a biomarker for prognostic stratification of patients with thin melanoma. *Biomark Res.* 2019;7:1.

31. Chen K, Zhao R, Yao G, Liu Z, Shi R, Geng J. Overexpression of kin of IRRE-Like protein 1 (KIRREL) as a prognostic biomarker for breast cancer. *Pathol Res Pract.* 2020;216(7):153000.

32. Chen J, Wang X, Hu B, He Y, Qian X, Wang W. Candidate genes in gastric cancer identified by constructing a weighted gene co-expression network. *PeerJ.* 2018;6:e4692.

33. Hu B, Shi C, Jiang HX, Qin SY. Identification of novel therapeutic target genes and pathway in pancreatic cancer by integrative analysis. *Medicine (Baltimore).* 2017;96(42):e8261.

34. Wang K, Gao M, Yang M, et al. Transcriptome analysis of bronchoalveolar lavage fluid from children with severe Mycoplasma pneumoniae pneumonia reveals novel gene expression and immunodeficiency. *Hum Genomics.* 2017;11(1):4.

35. Huang CR, Chang TW, Lee CT, Shen CJ, Chang WC, Chen BK. ARNT deficiency represses pyruvate dehydrogenase kinase 1 to trigger ROS production and melanoma metastasis. *Oncogenesis.* 2021;10(1):11.

36. Goracy J, Bogacz A, Uzar I, et al. The Analysis of NADPH Quinone Reductase 1 (NQO1) Polymorphism in Polish Patients with Colorectal Cancer. *Biomolecules.* 2021;11(7).

37. Jiang ZN, Ahmed SMU, Wang QC, Shi HF, Tang XW. Quinone oxidoreductase 1 is overexpressed in gastric cancer and associated with outcome of adjuvant chemotherapy and survival. *World J Gastroenterol.* 2021;27(22):3085-3096.

38. Totten SP, Im YK, Cepeda Canedo E, et al. STAT1 potentiates oxidative stress revealing a targetable vulnerability that increases phenformin efficacy in breast cancer. *Nat Commun.* 2021;12(1):3299.

39. Tian X, Liu Y, Wang H, et al. The role of miR-199b-3p in regulating Nrf2 pathway by dihydromyricetin to alleviate septic acute kidney injury. *Free Radic Res.* 2021:1-11.

40. Li WC, Yao SP, Zhang J, Liu WB, Liu J, Geng CK. Low-dose lipopolysaccharide protects nerve cells against spinal cord injury via regulating the PI3K-AKT-Nrf2 signaling pathway. *Biochem Cell Biol.* 2021:1-9.

41. Faborode OS, Dalle E, Mabandla MV. Exposure to footshock stress downregulates antioxidant genes and increases neuronal apoptosis in an Abeta(1-42) rat model of Alzheimer's disease. *Neurochem Int.* 2021;150:105170.

42. Li T, Zhang C, Zhao G, et al. IGFBP2 regulates PD-L1 expression by activating the EGFR-STAT3 signaling pathway in malignant melanoma. *Cancer Lett.* 2020;477:19-30.

43. Zhao S, Wu L, Kuang Y, et al. Downregulation of CD147 induces malignant melanoma cell apoptosis via the regulation of IGFBP2 expression. *Int J Oncol.* 2018;53(6):2397-2408.

44. Strub T, Ghiraldini FG, Carcamo S, et al. SIRT6 haploinsufficiency induces BRAF(V600E) melanoma cell resistance to MAPK inhibitors via IGF signalling. *Nat Commun.* 2018;9(1):3440.

45. Yuan Q, Wang SQ, Zhang GT, et al. Highly expressed of SERPINA3 indicated poor prognosis and involved in immune suppression in glioma. *Immun Inflamm Dis.* 2021.

46. Nikolaev AA, Babkina IV, Gershtein ES, et al. Prognostic significance of the TNM system criteria, levels of serum insulin-like growth factors and their transport proteins, VEGF and MMP-7 in colorectal cancer. *Klin Lab Diagn.* 2021;66(8):459-464.

47. Aziz MNM, Rahim NFC, Hussin Y, et al. Anti-Metastatic and Anti-Angiogenic Effects of Curcumin Analog DK1 on Human Osteosarcoma Cells In Vitro. *Pharmaceuticals (Basel).* 2021;14(6).

48. Lau ES, Paniagua SM, Zarbafian S, et al. Cardiovascular Biomarkers of Obesity and Overlap With Cardiometabolic Dysfunction. *J Am Heart Assoc.* 2021;10(14):e020215.

49. Czogala W, Strojny W, Tomasik P, et al. The Insight into Insulin-Like Growth Factors and Insulin-Like Growth-Factor-Binding Proteins and Metabolic Profile in Pediatric Obesity. *Nutrients.* 2021;13(7).

50. Zhang Y, Gao S, Liang K, et al. Exendin-4 gene modification and microscaffold encapsulation promote self-persistence and antidiabetic activity of MSCs. *Sci Adv.* 2021;7(27).

51. Qiu C, Duan Y, Wang B, et al. Serum Anti-PDLIM1 Autoantibody as Diagnostic Marker in Ovarian Cancer. *Front Immunol.* 2021;12:698312.

52. Wang H, Wang X, Xu L, Lin Y, Zhang J, Cao H. Low expression of CDHR1 is an independent unfavorable prognostic factor in glioma. *J Cancer.* 2021;12(17):5193-5205.

53. Li LM, Luo FJ, Song X. MicroRNA-370-3p inhibits cell proliferation and induces chronic myelogenous leukaemia cell apoptosis by suppressing PDLIM1/Wnt/beta-catenin signaling. *Neoplasma.* 2020;67(3):509-518.

54. Wan W, Long Y, Jin X, et al. Protective Role of microRNA-200a in Diabetic Retinopathy Through Downregulation of PDLIM1. *J Inflamm Res.* 2021;14:2411-2424.

55. Milanesi E, Manda G, Dobre M, et al. Distinctive Under-Expression Profile of Inflammatory and Redox Genes in the Blood of Elderly Patients with Cardiovascular Disease. *J Inflamm Res.* 2021;14:429-442.

56. Hayashi G, Cortopassi G. Lymphoblast Oxidative Stress Genes as Potential Biomarkers of Disease Severity and Drug Effect in Friedreich's Ataxia. *PLoS One.* 2016;11(4):e0153574.

57. Zhao L, Liu H, Luo S, et al. Associations between genetic variants of KIF5B, FMN1, and MGAT3 in the cadherin pathway and pancreatic cancer risk. *Cancer Med.* 2020;9(24):9620-9631.

58. Jaeger E, Webb E, Howarth K, et al. Common genetic variants at the CRAC1 (HMPS) locus on chromosome 15q13.3 influence colorectal cancer risk. *Nat Genet.* 2008;40(1):26-28.

59. Al-Qattan MM, Alkuraya FS. Cenani-Lenz syndrome and other related syndactyly disorders due to variants in LRP4, GREM1/FMN1, and APC: Insight into the pathogenesis and the relationship to polyposis through the WNT and BMP antagonistic pathways. *Am J Med Genet A.* 2019;179(2):266-279.

60. Zagajewska K, Piatkowska M, Goryca K, et al. GWAS links variants in neuronal development and actin remodeling related loci with pseudoexfoliation syndrome without glaucoma. *Exp Eye Res.* 2018;168:138-148.

61. Giacopuzzi E, Gennarelli M, Minelli A, et al. Exome sequencing in schizophrenic patients with high levels of homozygosity identifies novel and extremely rare mutations in the GABA/glutamatergic pathways. *PLoS One.* 2017;12(8):e0182778.

62. Wang H, Hu Q, Tong Y, et al. LncRNA SOX2-OT regulates miR-192-5p/RAB2A axis and ERK pathway to promote glioblastoma cell growth. *Cell Cycle.* 2021:1-11.

63. Sun X, Hu X, Wang X, Jiang X. MiR-381-3p/RAB2A axis activates cell proliferation and inhibits cell apoptosis in bladder cancer. *Cell Mol Biol (Noisy-le-grand).* 2020;66(6):117-120.

64. Zhao F, Zhong M, Pei W, Tian B, Cai Y. miR-376c-3p modulates the properties of breast cancer stem cells by targeting RAB2A. *Exp Ther Med.* 2020;20(5):68.

65. Wang X, Yu Y, Huang Y, et al. Identification of potential diagnostic gene biomarkers in patients with osteoarthritis. *Sci Rep.* 2020;10(1):13591.

66. Takata A, Ionita-Laza I, Gogos JA, Xu B, Karayiorgou M. De Novo Synonymous Mutations in Regulatory Elements Contribute to the Genetic Etiology of Autism and Schizophrenia. *Neuron.* 2016;89(5):940-947.

67. Puthiyedth N, Riveros C, Berretta R, Moscato P. Identification of Differentially Expressed Genes through Integrated Study of Alzheimer's Disease Affected Brain Regions. *PLoS One.* 2016;11(4):e0152342.

68. Kaochar S, Dong J, Torres M, et al. ICG-001 Exerts Potent Anticancer Activity Against Uveal Melanoma Cells. *Invest Ophthalmol Vis Sci.* 2018;59(1):132-143.

69. Kahm YJ, Kim RK, Jung U, Kim IG. Epithelial membrane protein 3 regulates lung cancer stem cells via the TGFbeta signaling pathway. *Int J Oncol.* 2021;59(4).

70. Huang C, Xiong Z, Yang Q, Li X. Systematic Analysis of 4-gene Prognostic Signature in Patients with Diffuse Gliomas Based on Gene Expression Profiles. *J Cancer.* 2021;12(14):4295-4306.

71. Li X, Dai Y, Chen B, Huang J, Chen S, Jiang L. Clinical significance of CD34(+)CD117(dim)/CD34(+)CD117(bri) myeloblast-associated gene expression in t(8;21) acute myeloid leukemia. *Front Med.* 2021;15(4):608-620.

72. Nielsen K, Heegaard S, Vorum H, Birkenkamp-Demtroder K, Ehlers N, Orntoft TF. Altered expression of CLC, DSG3, EMP3, S100A2, and SLPI in corneal epithelium from keratoconus patients. *Cornea.* 2005;24(6):661-668.

73. Xu Y, Xu WH, Yang XL, Zhang HL, Zhang XF. Fatty acid-binding protein 5 predicts poor prognosis in patients with uveal melanoma. *Oncol Lett.* 2020;19(3):1771-1780.

74. Xu Y, Han W, Xu WH, et al. Identification of differentially expressed genes and functional annotations associated with metastases of the uveal melanoma. *J Cell Biochem.* 2019;120(11):19202-19214.

75. Han W, Xu WH, Wang JX, et al. Identification, Validation, and Functional Annotations of Genome-Wide Profile Variation between Melanocytic Nevus and Malignant Melanoma. *Biomed Res Int.* 2020;2020:1840415.

76. Wasinger C, Kunzl M, Minichsdorfer C, Holler C, Zellner M, Hohenegger M. Autocrine secretion of 15d-PGJ2 mediates simvastatin-induced apoptotic burst in human metastatic melanoma cells. *Br J Pharmacol.* 2014;171(24):5708-5727.

77. Faller WJ, Rafferty M, Hegarty S, et al. Metallothionein 1E is methylated in malignant melanoma and increases sensitivity to cisplatin-induced apoptosis. *Melanoma Res.* 2010;20(5):392-400.

78. Chen J, Alduais Y, Zhang K, Zhu X, Chen B. CCAT1/FABP5 promotes tumour progression through mediating fatty acid metabolism and stabilizing PI3K/AKT/mTOR signalling in lung adenocarcinoma. *J Cell Mol Med.* 2021.

79. Wang W, Liu Z, Chen X, et al. Downregulation of FABP5 Suppresses the Proliferation and Induces the Apoptosis of Gastric Cancer Cells Through the Hippo Signaling Pathway. *DNA Cell Biol.* 2021;40(8):1076-1086.

80. De Mattos-Arruda L, Cortes J, Blanco-Heredia J, et al. The temporal mutational and immune tumour microenvironment remodelling of HER2-negative primary breast cancers. *NPJ Breast Cancer.* 2021;7(1):73.

81. He YL, Chen MT, Wang T, et al. Development of FABP4/5 inhibitors with potential therapeutic effect on type 2 Diabetes Mellitus. *Eur J Med Chem.* 2021;224:113720.

82. Kozlowska D, Mysliwiec H, Harasim-Symbor E, Milewska AJ, Chabowski A, Flisiak I. Serum fatty acid binding protein 5 (FABP5) as a potential biomarker of inflammation in psoriasis. *Mol Biol Rep.* 2021;48(5):4421-4429.

83. Guo Q, Kawahata I, Degawa T, et al. Fatty Acid-Binding Proteins Aggravate Cerebral Ischemia-Reperfusion Injury in Mice. *Biomedicines.* 2021;9(5).

84. Tsantoulis P, Delorenzi M, Bieche I, et al. Prospective validation in epithelial tumors of a gene expression predictor of liver metastasis derived from uveal melanoma. *Sci Rep.* 2019;9(1):17178.

85. Duciel L, Anezo O, Mandal K, et al. Protein tyrosine phosphatase 4A3 (PTP4A3/PRL-3) promotes the aggressiveness of human uveal melanoma through dephosphorylation of CRMP2. *Sci Rep.* 2019;9(1):2990.

86. Maacha S, Anezo O, Foy M, et al. Protein Tyrosine Phosphatase 4A3 (PTP4A3) Promotes Human Uveal Melanoma Aggressiveness Through Membrane Accumulation of Matrix Metalloproteinase 14 (MMP14). *Invest Ophthalmol Vis Sci.* 2016;57(4):1982-1990.

87. Johansson JA, Marie KL, Lu Y, et al. PRL3-DDX21 Transcriptional Control of Endolysosomal Genes Restricts Melanocyte Stem Cell Differentiation. *Dev Cell.* 2020;54(3):317-332 e319.

88. Csoboz B, Gombos I, Tatrai E, et al. Chemotherapy induced PRL3 expression promotes cancer growth via plasma membrane remodeling and specific alterations of caveolae-associated signaling. *Cell Commun Signal.* 2018;16(1):51.

89. Foy M, Anezo O, Saule S, Planque N. PRL-3/PTP4A3 phosphatase regulates integrin beta1 in adhesion structures during migration of human ocular melanoma cells. *Exp Cell Res.* 2017;353(2):88-99.

90. Smith CN, Blackburn JS. PRL-3 promotes a positive feedback loop between STAT1/2-induced gene expression and glycolysis in multiple myeloma. *FEBS J.* 2021.

91. Lazo JS, Sharlow ER, Cornelison R, et al. Credentialing and Pharmacologically Targeting PTP4A3 Phosphatase as a Molecular Target for Ovarian Cancer. *Biomolecules.* 2021;11(7).

92. Liu C, Zhong W, Xia L, Fang C, Liu H, Liu X. A retrospective cohort study of clinical value of PRL-3 in stage III human colorectal cancer. *Medicine (Baltimore).* 2021;100(17):e25658.

93. Bersini S, Arrojo EDR, Huang L, Shokhirev MN, Hetzer MW. Transcriptional and Functional Changes of the Human Microvasculature during Physiological Aging and Alzheimer Disease. *Adv Biosyst.* 2020;4(5):e2000044.

94. Luo H, Ma C. Identification of prognostic genes in uveal melanoma microenvironment. *PLoS One.* 2020;15(11):e0242263.

95. Xing Q, Liu S, Luan J, Wang Y, Ma L. A novel 13 RNA binding proteins (RBPs) signature could predict prostate cancer biochemical recurrence. *Pathol Res Pract.* 2021;225:153587.

96. Zhao Y, Zhou H, Dong W. LncRNA RHPN1-AS1 promotes the progression of nasopharyngeal carcinoma by targeting CELF2 expression. *Exp Mol Pathol.* 2021;122:104671.

97. Wang L, Liu Z, Liu L, et al. CELF2 is a candidate prognostic and immunotherapy biomarker in triple-negative breast cancer and lung squamous cell carcinoma: A pan-cancer analysis. *J Cell Mol Med.* 2021;25(15):7559-7574.

98. El Bitar F, Al Sudairy N, Qadi N, et al. A Comprehensive Analysis of Unique and Recurrent Copy Number Variations in Alzheimer's Disease and its Related Disorders. *Curr Alzheimer Res.* 2020;17(10):926-938.

99. Peng G, Su J, Xiao S, Liu Q. LINC00152 acts as a potential marker in gliomas and promotes tumor proliferation and invasion through the LINC00152/miR-107/RAB10 axis. *J Neurooncol.* 2021.

100. Cui C, Wang Y, Gong W, et al. Long Non-Coding RNA LINC00152 Regulates Self-Renewal of Leukemia Stem Cells and Induces Chemo-Resistance in Acute Myeloid Leukemia. *Front Oncol.* 2021;11:694021.

101. Li Q, Wang X, Zhou L, et al. A Positive Feedback Loop of Long Noncoding RNA LINC00152 and KLF5 Facilitates Breast Cancer Growth. *Front Oncol.* 2021;11:619915.

102. Zhang J, Gao FF, Xie J. LncRNA linc00152/NF-kappaB feedback loop promotes fibroblast-like synovial cells inflammation in rheumatoid arthritis via regulating miR-103a/TAK1 axis and YY1 expression. *Immun Inflamm Dis.* 2021;9(3):681-693.

103. Yang PP, Yu XH, Zhou J. Tryptophanyl-tRNA synthetase (WARS) expression in uveal melanoma - possible contributor during uveal melanoma progression. *Biosci Biotechnol Biochem.* 2020;84(3):471-480.

104. Peters MAM, Meijer C, Fehrmann RSN, et al. Serotonin and Dopamine Receptor Expression in Solid Tumours Including Rare Cancers. *Pathol Oncol Res.* 2020;26(3):1539-1547.

105. Le-Bel G, Benhassine M, Landreville S, Guerin SL. Analysis of the proteasome activity and the turnover of the serotonin receptor 2B (HTR2B) in human uveal melanoma. *Exp Eye Res.* 2019;184:72-77.

106. Li Y, Yao Q, Zhang L, et al. Immunohistochemistry-Based Consensus Molecular Subtypes as a Prognostic and Predictive Biomarker for Adjuvant Chemotherapy in Patients with Stage II Colorectal Cancer. *Oncologist.* 2020;25(12):e1968-e1979.

107. Jiang SH, Li J, Dong FY, et al. Increased Serotonin Signaling Contributes to the Warburg Effect in Pancreatic Tumor Cells Under Metabolic Stress and Promotes Growth of Pancreatic Tumors in Mice. *Gastroenterology.* 2017;153(1):277-291 e219.

108. Henriksen R, Dizeyi N, Abrahamsson PA. Expression of serotonin receptors 5-HT1A, 5-HT1B, 5-HT2B and 5-HT4 in ovary and in ovarian tumours. *Anticancer Res.* 2012;32(4):1361-1366.

109. Wang X, Li H, Zhang A, et al. Diversity among Differentially Expressed Genes in Atrial Appendages of Atrial Fibrillation: The Role and Mechanism of SPP1 in Atrial Fibrosis. *Int J Biochem Cell Biol.* 2021:106074.

110. Lu X, Fan Y, Li M, Chang X, Qian J. HTR2B and SLC5A3 Are Specific Markers in Age-Related Osteoarthritis and Involved in Apoptosis and Inflammation of Osteoarthritis Synovial Cells. *Front Mol Biosci.* 2021;8:691602.

111. Jin B, Ha SE, Wei L, et al. Colonic Motility Is Improved by the Activation of 5-HT2B Receptors on Interstitial Cells of Cajal in Diabetic Mice. *Gastroenterology.* 2021;161(2):608-622 e607.
